# Comparison of Pathogenicity-Related Genes in the Current Pseudorabies Virus Outbreak in China

**DOI:** 10.1038/s41598-017-08269-3

**Published:** 2017-08-10

**Authors:** Yan-Dong Tang, Ji-Ting Liu, Tong-Yun Wang, Ming-Xia Sun, Zhi-Jun Tian, Xue-Hui Cai

**Affiliations:** 1 State Key Laboratory of Veterinary Biotechnology, Harbin Veterinary Research Institute of Chinese Academy of Agricultural Sciences, Harbin, 150001 China; 2 College of Animal Science and Technology, Jilin Agriculture University, Changchun, 130018 China

## Abstract

There is currently a pandemic of pseudorabies virus (PRV) variant strains in China. Despite extensive research on PRV variant strains in the past two years, few studies have investigated PRV pathogenicity-related genes. To determine which gene(s) is/are linked to PRV virulence, ten putative virulence genes were knocked out using clustered regularly interspaced palindromic repeats (CRISPR)/Cas9 technology. The pathogenicity of these mutants was evaluated in a mouse model. Our results demonstrated that of the ten tested genes, the thymidine kinase (TK) and glycoprotein M (gM) knockout mutants displayed significantly reduced virulence. However, mutants of other putative virulence genes, such as glycoprotein E (gE), glycoprotein I (gI), Us2, Us9, Us3, glycoprotein G (gG), glycoprotein N (gN) and early protein 0 (EP0), did not exhibit significantly reduced virulence compared to that of the wild-type PRV. To our knowledge, this study is the first to compare virulence genes from the current pandemic PRV variant strain. This study will provide a valuable reference for scientists to design effective live attenuated vaccines in the future.

## Introduction

Pseudorabies virus (PRV) is a swine alpha-herpesvirus that causes considerable economic losses to the swine industry^1^. In China, PRV has been well controlled for decades by the Bartha-K61 vaccine, and it was thought that this disease would be eradicated in the foreseeable future. Despite great efforts to promote PRV vaccination, an unprecedented large-scale outbreak of PRV variants in China has caused great economic losses to the Chinese swine industry^2, 3^. These re-emerging pseudorabies variants belong to genotype II, and their sequences exhibit significant differences from genotype I^4, 5^. Compared to classic virulent PRV strains, these variant strains exhibit increased virulence, with an earlier onset of clinical signs and higher mortality in swine^5, 6^. Although several studies have focused on PRV variant strains in the past two years, few studies have investigated PRV pathogenicity-related genes. This information will be valuable for controlling this re-emerging pathogen.

Because of its large genome, PRV has traditionally been genetically manipulated using bacterial artificial chromosome (BAC) techniques^7^. However, BAC mutagenesis is available only for virus isolates for which a useful BAC has been produced. In addition, during BAC system construction, the insertion of selection markers or parts of BAC plasmids into the viral genome may affect viral function. The clustered regularly interspaced palindromic repeats (CRISPR)/associated (Cas9) system was a revolutionary development in gene-editing technology^8, 9^. CRISPR/Cas9 can specifically break the targeted DNA with high efficiency and thereby cause indels in the target region via nonhomologous end joining (NHEJ) DNA damage repair or via homologous directed repair (HDR) in the presence of a homologous DNA donor^8, 10^. This method is simple, requiring only the design of an effective single-guide RNA (sgRNA) that is specific to a given target gene. Large genomic DNA viruses, such as PRV, can be edited easily, and the only delay involves determining the targeted sequence^11–15^.

In this study, we knocked out ten putative pathogenicity-related genes (glycoprotein E (gE), glycoprotein I (gI), Us2, Us9, thymidine kinase (TK), Us3, early protein 0 (EP0), glycoprotein M (gM), glycoprotein G (gG) and glycoprotein N (gN)) and compared the virulence of different mutants in a sensitive mouse model. We chose gE, gI, Us2 and Us9 because a classical live attenuated vaccine, Bartha-K, featured a large deletion that included these four genes^16, 17^. Additionally, TK, Us3, and EP0 have been reported to be correlated with classical PRV virulence^18–21^. We chose the envelope proteins gM, gG and gN because these genes have potential in the DIVA (differentiating infected from vaccinated animals) strategy. This study uncovered pathogenicity-related genes of re-emerging PRV variants for the first time and will provide a valuable reference for the control of PRV variants.

## Materials and Methods

### Cell lines and viruses

Vero cells were cultured in DMEM with 10% FBS and streptomycin/penicillin. The PRV HeN1 strain was the first PRV variant strain isolated in China and was isolated in our laboratory (GenBank accession number: KP098534.1). The properties of the HeN1 strain have been described previously^2, 4^. The Us2, Us3, Us9 and gE/gI knockout PRV strains were described in our previous reports^12, 13^.

### Generation of CRISPR/Cas9 sgRNA constructs

Specific gene-targeted sgRNAs were designed using an online CRISPR Design Tool (https://wwws.blueheronbio.com/external/tools/gRNASrc.jsp), and the target regions were primarily located downstream of the start codons of the coding regions of specific genes. Generally, four sgRNAs were designed for each gene, and we selected only the most effective sgRNA for further specific gene knockout. The effectiveness of the sgRNAs was screened with a firefly luciferase-tagged recombinant virus, as described in our previous report^13^. Using this virus, if the sgRNA targets the PRV genome, the genomic DNA will be broken by Cas9, and luciferase expression will thereby decrease accordingly. The most effective sgRNAs identified after screening are listed in Table 1.

**Table 1:** sgRNAs used in this study.

sgRNA-gG	5′-CACCGCCTCGCCCTCGGGCTCCTCG-3′
	5′-AAACCGAGGAGCCCGAGGGCGAGGC-3′
sgRNA-gM	5′-CACCGGCAACGCCGAGGCCGTGAGC-3′
	5′-AAACGCTCACGGCCTCGGCGTTGCC-3′
sgRNA-gN	5′-CACCGCTCTTCCATAGTCTTTTCCG-3′
	5′-AAACCGGAAAAGACTATGGAAGAGC-3′
sgRNA-TK	5′-CACCGCATCAGCGCGGCGGCCTTCG-3′
	5′-AAACCGAAGGCCGCCGCGCTGATGC-3′
sgRNA-EP0	5′-CACCGTCTGGACGTCGCGGCCACCG-3′
	5′-AAACCGGTGGCCGCGACGTCCAGAC-3′

### PRV gene knockout using CRISPR/Cas9 technology

The gene knockout procedure was similar to that described in our previous reports^12, 13^. First, Vero cells were seeded in 12-well plates and transiently transfected 12 h later with the indicated CRISPR/Cas9 plasmids (2 μg per well). Then, 12 h post-transfection, PRV HeN1 was inoculated at an MOI of 0.01. At 48 h post-infection (hpi), the supernatants were collected for plaque purification. Several plaques were selected randomly for viral DNA extraction, and gene knockout was confirmed via DNA sequencing and western blot. The western blot procedure was similar as described previously^12^. The PCR primers used to assess gene knockout are listed in Table 2.

**Table 2:** PCR primers used in this study.

gG-F	5′-ACCGCTACGACACCAAGGTC-3′
gG-R	5′-GCCGCCGTCAAAGAACCAG-3′
gN-F	5′-TACAATCGCCTGCACCTCGC-3′
gN-R	5′-AGGAGCCGTGGCCATCGTAG-3′
gM-F	5′-AAGAAGCTGGTCACGGTGGG-3′
gM-R	5′-AGCTGCGCGTTGATCGTGGC-3′
TK-F	5′-AAGCAGAACGGCAGCCTGAGCG-3′
TK-R	5′-GGGCACGGCAAACTTTATTGGGAT-3′
EP0-F	5′-CGCAGCGCCGCT TTCAGACCCA-3′
EP0-R	5′-GGAGCATGGCC TCGGTCAC-3′

### Plaque purification

Vero cells were seeded in 60-mm dishes, and the indicated viruses were collected and subjected to serial 10-fold dilutions for the plaque-forming assay. At 2 hpi, the cells were washed three times with PBS and overlaid with 2% low-melting-point agarose (Lonza, USA) in DMEM medium containing 2% FBS. The dishes were further incubated at 37 °C for 3–5 days to allow plaques to form, and a single plaque was then purified.

### *In vitro* growth properties of mutant viruses

Vero cells were infected with wild-type PRV HeN1 or the indicated gene knockout PRV mutants at an MOI of 0.01. The infected cells were collected at 12, 24, 36 and 48 hpi. Serial 10-fold dilutions of the indicated viruses were used to infect Vero cells. The viral titers at different times were recorded as the 50% tissue culture infection dose (TCID50).

### Analysis of the virulence of mutant viruses

Six- to eight-week-old female SPF BALB/c mice were divided into 12 groups (5 mice per group), and each group was infected with HeN1 or the indicated PRV mutant via subcutaneous injection with 2 × 10^4^ PFUs of virus or DMEM (100 μL). The results from two independent experiments are presented as two experiments together. All animal experiments were approved by the Animal Ethics Committee of Harbin Veterinary Research Institute of the Chinese Academy of Agricultural Sciences and were performed in accordance with animal use ethical guidelines and approved protocols.

### Statistical analysis

Statistical analyses were performed using an ANOVA, as implemented in SPSS version 19.0. P < 0.05 was considered statistically significant.

## Results

### Knockout and replicative properties of ten putative virulence genes

**Figure 1:**
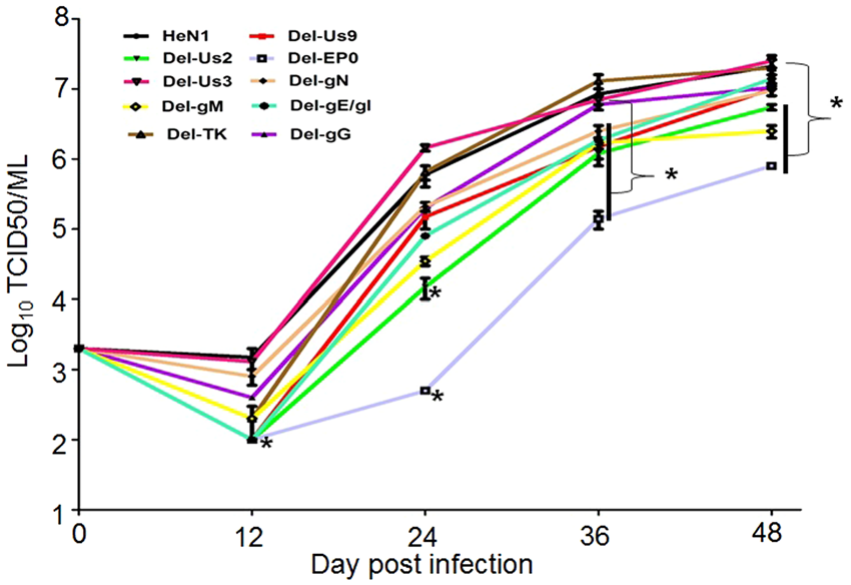
Replication kinetics of knockout viruses. Vero cells were infected with wild-type PRV HeN1 or the indicated gene knockout PRV mutants at an MOI of 0.01. The infected cells were collected at 12, 24, 36 and 48 hpi. The viral titers at different times were recorded as the 50% tissue culture infection dose (TCID50).

In this study, we evaluated ten putative pathogenicity-related genes in mice. To create the indicated mutants, we applied CRISPR/Cas9 technology to rapidly knock out putative virulence gene(s). First, we designed sgRNAs and screened for effective sgRNAs, as described previously^12–14^. Second, we used the most effective sgRNA to knock out the corresponding gene. Finally, the sgRNA-treated PRV was purified via several rounds of plaque purification. The PRV mutations were confirmed via DNA sequencing (Fig. S), and the CRISPR/Cas9 knockout efficiency for all ten genes was determined, as shown in Table S. Western blot further confirmed that gE, gI, Us3, and EP0 were successfully knocked out (Fig. S (F to H)). For the TK knockout, it was previously reported that acyclovir could inhibit PRV containing an intact TK gene. Therefore, in this study, we used acyclovir to evaluate whether TK was successfully knocked out. As shown in Fig. S (I), acyclovir inhibited wild-type viral replication and had no effect on the triple-mutant TK HeN1 strain. For other genes, no antibody was available, so we were unable to examine these mutants at the protein level. However, it is known that CRISPR/Cas9 targeting of the 3′ region of an ORF will potentially result in the expression of truncated products, whereas targeting of the 5′ ORF will create alternative ATG translation start sites with the potential to produce protein products. In our study, we designed sgRNAs targeting the 5′ ORF. When we further analyzed alternative ATG translation start sites with the potential to produce protein products, we found only very small ORFs, which we do not believe could produce functional proteins. After the desired mutants were successfully prepared, we tested whether the replication of these mutants was influenced by the knockout of the indicated gene(s) *in vitro*. Our results indicated that the TK and Us3 null mutants had replication kinetics similar to wild-type PRV; however, other mutations affected viral replication to different degrees (Fig. 1). Of all the investigated mutants, the EP0-negative mutant replicated at the slowest rate (Fig. 1). This finding indicates that EP0 gene products are very important for viral replication.

### Pathogenic evaluation of ten putative virulence gene knockout mutants in mice

**Figure 2:**
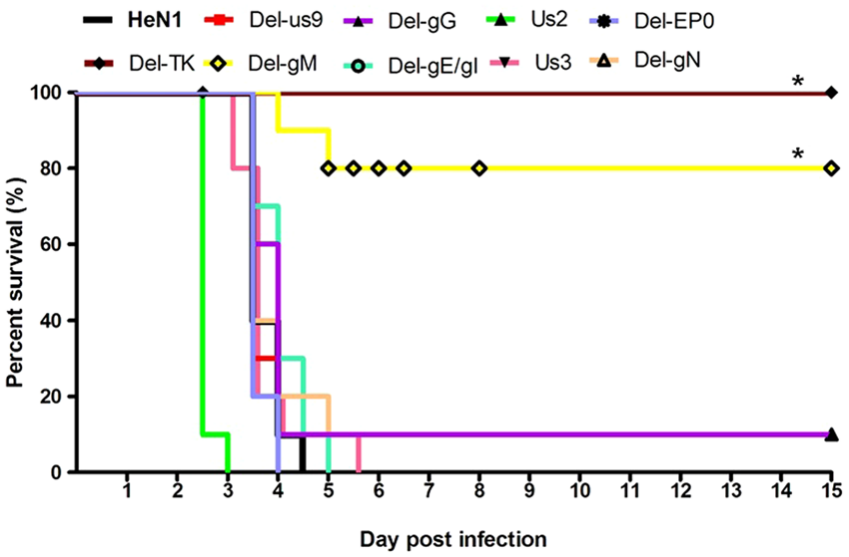
Pathogenicity of the indicated PRV mutants. Six- to eight-week-old female SPF BALB/c mice were divided into 11 groups, and each group was infected with HeN1 or the indicated PRV mutant via subcutaneous injection with 2 × 10^4^ PFUs of each virus or DMEM (100 μL). Survival was recorded for 15 days.

To investigate whether these putative virulence genes were critical for PRV pathogenicity, we used a susceptible mouse model. Although the TK null mutant replicated similarly to the wild-type PRV *in vitro*, this mutant completely lost its pathogenicity (Fig. 2). No mice died, and no clinical signs were observed in the TK groups. The gM gene was also significantly associated with virulence: only two of ten mice infected with mutants for this gene died (Fig. 2). The two dead mice manifested clinical signs similar to those observed in mice infected with wild-type PRV, but the clinical signs were delayed compared to those in the wild-type PRV. Most of the mutants, such as the gE, gI, Us9, Us3, gG, gN and EP0 mutants, exhibited no significant virulence reduction compared to that of the wild-type PRV (Fig. 2). However, interestingly, the Us2 mutant caused a more rapid onset of symptoms and earlier death than the wild-type PRV (Fig. 2).

## Discussion

In this study, we chose sensitive mice to evaluate PRV pathogenesis. Among all PRV hosts, pigs are known to be the most insensitive to PRV infection, while mice and sheep are more sensitive to PRV infection. Thus, the use of pigs may not reveal a pathogenic difference. In another study that supports our opinions, mice infected with a gE/gI deletion mutant displayed high morbidity and mortality, whereas infected pigs remained clinically normal^22^. A similar result was achieved in a sheep experiment^22^. Another group in our laboratory also performed a pathogenicity experiment with gE/gI and TK deletion mutants. After being infected with these two mutants, all the pigs remained clinically normal, and no adverse reactions were observed (unpublished data). Therefore, we think that mice are more susceptible to PRV infection than pigs, and mice may be more suitable for evaluating the pathogenicity of PRV.

The TK gene is found only within *Alphaherpesvirinae* and *Gammaherpesvirinae*. This gene differs from cellular TK genes and plays a critical role in the synthesis of dTTP^23^. For classical PRV strains, TK-negative PRV mutants were highly attenuated in mice, rabbits and pigs and conferred protective immunity against PRV challenge in pigs^18, 19^. Consistent with classical PRV strains, the TK gene was also an important virulent gene in the re-emerging PRV variant. In mice sensitive to PRV infection, the TK knockout mutant completely lost its pathogenicity. This finding indicates that future vaccine development against PRV variants should better inactivate this gene.

gM is a nonessential glycoprotein that is conserved throughout the herpesvirus family. gM was found to inhibit PRV-induced membrane fusion by altering the membrane trafficking itineraries^24, 25^. gM and gN can form a disulfide-linked complex, and the gM mutant exhibited significantly decreased plaque size without impaired viral penetration^26, 27^. gM was also shown to be involved in different steps during virus secondary envelopment^28^. The gM mutant of the classical PRV strain exhibited significantly impaired viral virulence in piglets and conferred protection against a challenge infection^29^. Our study also showed that, among all the tested genes, the role of gM in virulence was second only to that of TK. More importantly, gM is an envelope protein, and the gM mutant can be used to differentiate infected from vaccinated animals, similar to gE-deleted vaccines. We propose that both the TK and gM knockout strains may represent promising candidate genetically marked vaccines for the future control of this re-emerging pathogen.

Since the late 1980s, the Bartha-K61 vaccine has been widely applied in China, resulting in effective control of PRV pandemics^16^. The Bartha-K61 vaccine was developed via multiple passages of a virulent field strain in cultured chicken cells and embryos^23^. Molecular and genetic analyses have identified a deletion of approximately 3 kb that encompasses Us8 (gE), Us9 and a large portion of Us7 (gI) and Us2^17, 30, 31^. gE, gI and Us9 are associated with neurovirulence and are required for efficient anterograde spread in the nervous system^32–35^. The virulence of the Us9 null mutant was reduced in a rat model of eye infection^32^. However, in this study, the gE/gI mutant and the Us9 mutant manifested a virulence level similar to that of wild-type PRV (Fig. 2). This difference may be a result of the use of mice that were more sensitive to PRV infection. In a previous report, the virulence of a gE/gI mutant was evaluated in mice, sheep and pigs, and the gE/gI mutant was shown to be avirulent in pigs but virulent in mice and sheep^22^. From a biosafety perspective, the gE/gI deletion alone failed to generate a safe vaccine; the strain must be further attenuated. A limited number of studies have focused on Us2, which is a virion tegument component that is prenylated in infected cells^36^. Us2 binds to extracellular signal-regulated kinase (ERK), inhibiting the activation of ERK^37^. No previous reports have addressed the role of Us2 in virulence. Interestingly, we show for the first time that the Us2 null mutant caused an earlier onset of symptoms and earlier death than the wild-type virus. Why the Us2 null mutant exhibited increased pathogenicity needs to be explored further.

The PRV Us3 gene encodes a serine/threonine protein kinase that functions in multiple processes, including anti-apoptosis responses^38, 39^, cytoskeletal reorganization^40, 41^ and MHC-I downregulation^42^. Us3 is not required for growth *in vitro*, and the Us3 null mutant is slightly attenuated in mice, with a delayed onset of symptoms compared to that of the wild-type virus^43^. However, the Us3 null mutant exhibits strongly reduced virulence in pigs compared to that of the wild-type PRV^20^. In our report, we also obtained similar results in mice. Whether the Us3 null mutant exhibits reduced virulence in pigs must be explored further.

EP0 is expressed as an early protein in the PRV lifecycle, and this protein transactivates viral promoters, such as IE180, TK and gG^44^. In previous reports, an EP0 null mutant replicated slowly, exhibited significantly reduced virulence and elicited strong protective immunity against a lethal PRV challenge^21, 45, 46^. EP0 knockout attenuates PRV without affecting its immunogenicity, and such mutants were thought to be desirable vaccine candidates for PRV^46^. Although the EP0 mutant replicated slowly *in vitro* in previous reports, our results showed that the EP0 mutant replicated more slowly than other mutants, whereas the pathogenicity of the EP0 mutant was equivalent to that of wild-type PRV. This finding indicates that EP0 is not a critical virulence gene for this re-emerging PRV. Our result differed from previous reports, possibly because the PRV variant exhibited increased pathogenicity and the EP0 knockout alone may have a minimal effect on virulence in mice.

gG is the most abundant PRV protein found in the supernatant of PRV-infected cell cultures. gG binds to chemokines with high affinity, which in turn inhibits chemokine function, suggesting a role for gG in immune evasion^47^. In our study, we showed that gG is not a virulence gene in mice. However, because gG is involved in immune evasion, a candidate vaccine that contains a gG knockout may exhibit enhanced immunogenicity.

In conclusion, we evaluated ten putative virulence genes of the PRV variant and found that TK and gM were critical virulence genes in sensitive animals. The information obtained in this study will provide a valuable reference for scientists to effectively control PRV in the future.

## Electronic supplementary material

Supplementary Information
