# Supplementary material for: Comparison of Pathogenicity-Related Genes in the Current Pseudorabies Virus Outbreak in China
Source: Sci Rep. 2017 Aug 10;7:7783. doi: 10.1038/s41598-017-08269-3 (PMC5552686; doi:10.1038/s41598-017-08269-3)
Supplement: Supplementary file 1 — Supplementary Information [file 41598_2017_8269_MOESM1_ESM.pdf]

Comparison of Pathogenicity-Related Genes in the Current Pseudorabies Virus Outbreak in China

Yan-Dong Tang[1], Ji-Ting Liu[1, 2], Tong-Yun Wang[1], Ming-Xia Sun[1], Zhi-Jun Tian[1], Xue-Hui Cai[1]*

Figure S1 Gene knockouts confirmation. From (A) to (E), the indicated mutants were confirmed by DNA sequencing. From (F) to (H), the indicated mutants were confirmed by western blot. (I), TK deletion mutant was confirmed by ACV.

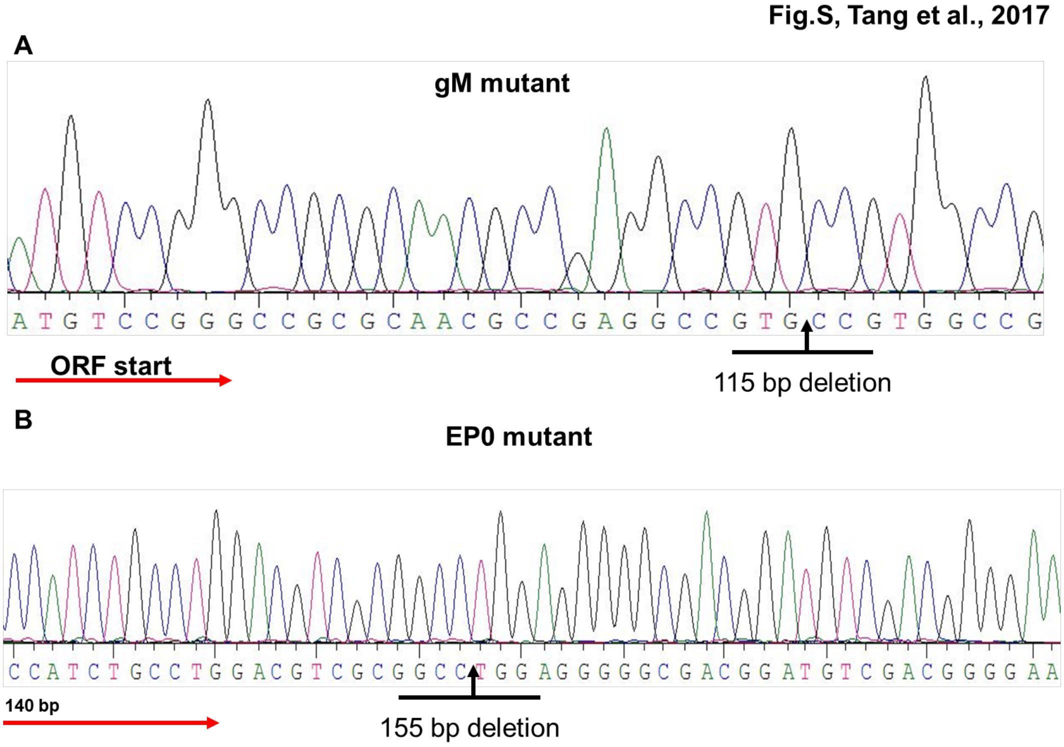

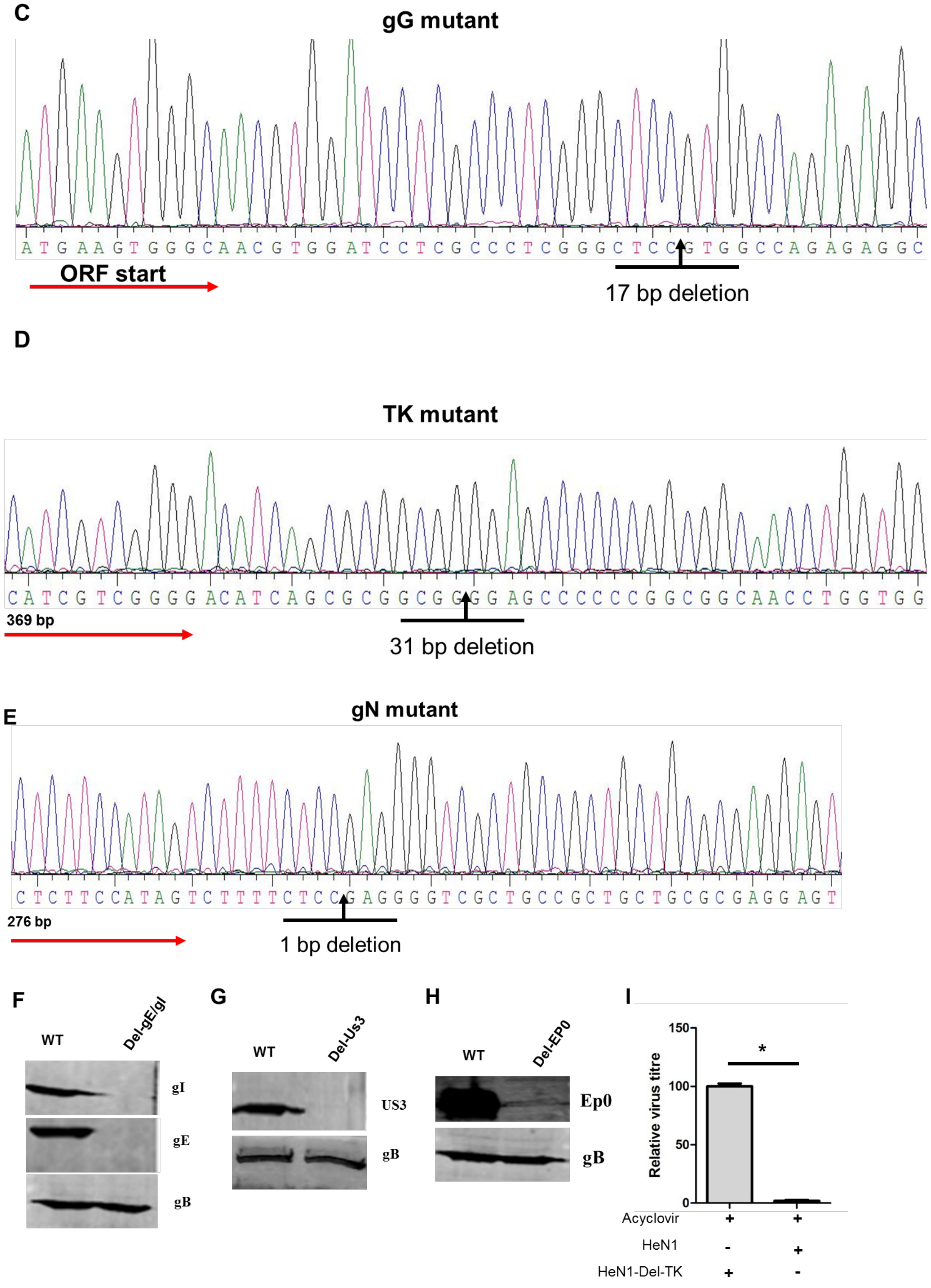

C
gG mutant
ATGAAGTGGGCAACGTGGATCCTCGCCCTCGGGCTCCGTGGCCAGAGAGGC
ORF start
17 bp deletion
D
TK mutant
CATCGTCGGGGACATCAGCGCGGCGCGGGAGCCCCCCGGCGGCAACCTGGTGG
369 bp
31 bp deletion
E
gN mutant
CTCTTCCATAGTCTTTTCTCCGAGGGGTCGCTGCCGCTGCTGCGCGAGGAGT
276 bp
1 bp deletion
F
WT
Del-gE/gI
gI
gE
gB
G
WT
Del-Us3
US3
gB
H
WT
Del-EP0
Ep0
gB
I
Relative virus titre
150
100
50
0
*
Acyclovir + +
HeN1 - +
HeN1-Del-TK + -

Table S. CRISPR/Cas9 knockout efficiency.

sgRNAs	Purified clones	Mutated clones	Mutated ratio
gRNA-gE	32	11	34.4%
gRNA-gI	12	2	16.7%
gRNA-Us2	7	3	42.9%
gRNA- Us3	12	4	33.3%
gRNA- Us9	8	2	25%
gRNA-EP0	23	4	17.4%
gRNA-TK	24	3	12.5%
gRNA-gG	12	4	33.3%
gRNA-gM	8	3	37.5%
gRNA-gN	11	2	18.2%
